# Retrospective detection for V-type OPNAs exposure via phosphonylation and disulfide adducts in albumin

**DOI:** 10.1038/s41598-022-15198-3

**Published:** 2022-06-29

**Authors:** Jin Wang, Fengjuan Sun, Xiaogang Lu, Runli Gao, Chengxin Pei, Hongmei Wang

**Affiliations:** State Key Laboratory of NBC Protection for Civilian, Beijing, 102205 China

**Keywords:** Natural hazards, Biomarkers

## Abstract

Organophosphorus nerve agents (OPNAs) that damage the central nervous system by inhibiting acetylcholinesterase activity, pose severe threats to human health and life security. Reliable biomarkers that quickly and accurately detect OPNAs exposure are urgently needed to help diagnose quickly and treat in time. Albumins that covalently bind to OPNAs could serve as important targets for retrospective verification of OPNAs exposure. The goal of this study is to explore the potential biomarkers in albumins with high reactivity and good stability and expand the group of potential biomarkers in different species for detecting the exposure of V-type OPNAs including O-ethyl S-(2-(diisopropylamino)ethyl) methylphosphonothioate (VX), O-isobutyl S-(2(diethylamino)ethyl) methylphosphonothioate (VR), and O-butyl S-(2-(diethylamino)ethyl) methylphosphonothioate (Vs). Taking human serum albumin (HSA), bovine serum albumin (BSA) and rabbit serum albumin (RSA) as the research objectives, multiple active sites including phosphonylation and disulfide adduct sites were observed in albumins from different species. Numerous phosphonylation sites labeled by all agents in one type of albumin were found. Among the different species, four shared phosphonylation sites with high reactivity include K499, K549, K249, and Y108. In addition, Y108 on ETY*GEMADCCAK, Y287 on Y*ICENQDSISSK, Y377 on TY*ETTLEK and Y164 on YLY*EIAR in HSA were stably phosphonylated by all agents in gradient concentration, making them stable and suitable potential biomarkers for V-type OPNAs exposure. Notably, Y108 on ETY*GEMADCCAK in HSA, on DTY*GDVADCCEK in RSA, and on ETY*GDMADCCEK in BSA were highly reactive to all V-type agents, regardless of species. It was also successfully labeled in HSA exposed to class V agents in gradient concentration. Y108 is expected to be used to screen and identify the exposure of V-type agents in the retrospective research. Disulfide adducts sites, consisted of four sites in HSA and two sites in BSA were also successfully labeled by V-type agents, and characteristic ion fragments from these disulfide adducts were also identified by secondary mass spectrometry. Molecular simulation of the stably modified sites were conducted to discover the promoting factors of covalent adduct formation, which help further clarify formation mechanism of albumin adducts at active sites.

## Introduction

Organophosphorus nerve agents (OPNAs), first synthesized in the mid-twentieth century, can cause nervous system dysfunction by irreversibly inhibiting acetylcholinesterase activity^1–5^. Once utilized as chemical warfare agents, OPNAs were banned by the Chemical Weapons Convention (CWC). However, they may still be exposed in various situations, such as terrorist attacks, armed conflicts and improper storage, which pose serious threats to human health, life security, environmental protection and world peace^2,3,6–8^. As a result, fast and accurate biomarkers are essential for detecting organophosphorus exposure^9–15^. Protein adducts, recognized as important targets for retrospective detection of OPNAs exposure, form via covalent bonding of the phosphorus atom in organophosphorus to active amino acids from the proteins^16–18^. Based on the principle of convenient sampling, butyrylcholinesterase (BChE) with high reactivity and albumin with high concentration in blood and easy covalent binding with OPNAs are the research focus of target proteins in the field^19–23^. However, OPNAs-BChE adducts face many disadvantages, such as complicated purification and easy aging. Thus, research has been increasingly directed toward OPNA- albumin adducts^24,25^.

As one of the important targets of OPNAs, albumin can form covalent adducts with various OPNAs^26–29^. OPNA-albumin adducts have large detection windows. The life cycle of albumin adducts is consistent with that of albumin. In addition, albumin adducts have high stability in blood. They are highly stable at multiple active sites, mainly involving tyrosine^30,31^, lysine^31–33^, and serine^34,35^. Tyr411 is an active site found on the surface of albumin, and it plays a pivotal role in confirming OPNAs exposure^36–38^. V-type OPNAs are known chemical warfare agents with high toxicity, including VX, Vs and VR^39–44^. Two types of adducts including phosphonylation and disulfide adducts can form in albumin exposed to class V agents^39,45,46^. Disulfide adducts generate when the sulfhydryl groups in the leaving group of V-type poisons form disulfide bonds with the sulfhydryl groups in the cysteine of a protein. Cys34 is the only amino acid that contains free sulfhydryl groups in albumin, and it can be easily modified by electrophilic agents^47^. In addition, diverse dipeptide or tripeptide adducts containing cysteine also can be acquired in albumin after V–type OPNA exposure^48,49^.

**Figure 1:**
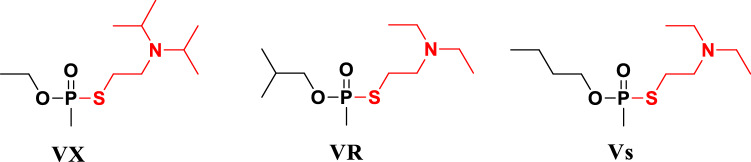
Structures of V-type OPNAs. Alkoxy phosphonic acid group is in black, and leaving group is in red.

In order to prevent the serious threats to human health and life safety after OPNAs exposure caused by unexpected events, it is essential to find stable and reliable protein adducts and expand the group of potential biomarkers in different species for retrospective detection of OPNAs exposure. This work study adducts in albumins with highly homologous sequences from different species (human, bovine and rabbit) exposed to V-type agents. Modified peptides were obtained following trypsin hydrolysis and then analyzed using the Orbitrap Fusion Lumos mass spectrometer. The structures of V-type OPNAs (i.e. VX, Vs, and VR) are shown in Fig. 1. Four phosphonylated sites modified by all class V agents were identified in albumins from different species, which emphasized the reliability of these adducts to some extent. Seven stably modified sites in HSA exposed to V-type OPNAs in gradient concentration were observed. Moreover, possible characteristic ions fragments of disulfide adduct were found in the mass spectra. Molecular docking between Vs and potential biomarkers of HSA was implemented to understand the possible promoting factors of adducts formation.

## Results and discussion

### Active sites modified by V-type agents

V-type OPNAs pose serious threats to human health and life safety^3,6,15,41,50,51^. The agents enter the body and attack albumin to form two types of protein adducts: phosphonylation adducts and disulfide adducts^40,45,52^. In order to expand the group of potential biomarkers in different species, the work utilized different albumins with similar amino acid sequences. The amino acid sequences in serum albumin from the UniProt database revealed that the sequence homologies of HSA with RSA and BSA were about 75.21% and 76.36%, respectively. Significant similarity among the active peptides indicated minimal differences in albumins from rabbits, bovines, and humans. Therefore, the modified sites shared by albumins from three species show the reliability of these adducts to a certain extent, providing reference data for finding potential biomarkers.

The active sites across all samples were modified by the V-type agents in two ways: phosphonylation and disulfide bond exchange. As shown in Tables 1, 2, 3, there were ten phosphonylation sites in BSA, eight in HSA and ten in RSA. These active sites were simultaneously labeled by the three poisons: Vs, VX and VR, indicating that the active sites had highly reactivity to these agents. However, it was different in the selectivity of OPNAs to active sites. For example, K438 on K*VPQVS*TPTLVEVSR in HSA could be modified by VX and Vs, while S443 could only be labeled with VR. K490 and S494 on LCVLHEK*TPVS*EK showed similar differences in selectivity. The differences in selection did not affect the productive information provided by the modified peptides after OPNAs exposure. Thus, the targeted identification of these modified peptides and active sites showed great potential to detecting the exposure of V-type agents.

**Table 1:** Phosphonylated sites modified by V-type agents in bovine albumin.

NO	Modified peptide	Binding sites	VX	Vs	VR
1	RPCFSALTPDETY*VPK	Y521	√	√	√
2	Y*NGVFQECCQAEDK	Y185	√	√	√
3	ETY*GDMADCCEK	Y108	√	√	√
4	LK*HLVDEPQNLIK	K402	√	√	√
5	GACLLPK*IETMR	K205	√	√	√
6	YICDNQDTISSK*LK	K298	√	√	√
7	FPK*AEFVEVTK	K249	√	√	√
8	LVTDLTK*VHK	K264	√	√	√
9	K*QTALVELLK	K549	√	√	√
10	FK*DLGEEHFK	K36	√	√	√

**Table 2:** Phosphonylated sites modified by V-type agents in human albumin.

NO	Modified peptide	Binding sites	VX	Vs	VR
1	TY*ETTLEK	Y377	√	√	√
2	Y*ICENQDSISSK	Y287	√	√	√
3	ETY*GEMADCCAK	Y108	√	√	√
4	YLY*EIAR	Y164	√	√	√
5	VTK*CCTESLVNR	K499	√	√	√
6	LDELRDEGK*ASSAK	K214	√	√	√
7	K*VPQVS*TPTLVEVSR	K438	√	√	
8		S443			√

**Table 3:** Phosphonylated sites modified by V-type agents in rabbit albumin.

NO	Modified peptide	Binding sites	VX	Vs	VR
1	VTK*CCSESLVDR	K499	√	√	√
2	K*QTALVELVK	K549	√	√	√
3	EK*ALISAAQER	K212	√	√	√
4	IVTDLTK*VHK	K264	√	√	√
5	Y*MCEHQETISSHLK	Y287	√	√	√
6	AY*EATLK	Y377	√	√	√
7	DTY*GDVADCCEK	Y108	√	√	√
8	LCVLHEK*TPVS*EK	K490		√	√
9		S494	√		
10	CCATDDPHACY*AK	Y394	√	√	√

Highly homologous sequences from the different species had the same phosphonylated peptides and active sites, even though some amino acid residues were different. As shown in Table 4, K499, K549, K249, and Y108 modified by poisons were shared active sites in HSA, BSA, and RSA. Remarkably, Y108 on ETY*GEMADCCAK in HSA, on DTY*GDVADCCEK in RSA and on ETY*GDMADCCEK in BSA, were phosphonylated by all the V-type agents. These active sites were deemed suitable as potential biomarkers for identifying V-type OPNAs exposure due to their low selectivity and high reactivity to class V toxicants. Furthermore, these sites showed no species differences. The other three modified sites exhibited different selectivity and reactivity to the OPNAs. These shared sites provided clear guides to act as biomarkers in the different species due to they could screen targeted V poisons. For example, K499 on VTK*CCTESLVNR could help screen the exposure of VR in BSA. Moreover, K249 on FPK*AEFVEVTK could be used to detect VR in BSA, and K549 on K*QTALVELVK could be used to identify VX in HSA. When these sites were located in albumins from other species, they could be used to screen for all V-type poisons.

**Table 4:** Phosphonylated shared sites in albumins from different species after V-type OPNAs exposure.

NO	Species	Modified sites	Phosphonylated peptide	VX	Vs	VR
1	HSA	K499	VTK*CCTESLVNR	√	√	√
	RSA		VTK*CCSESLVDR	√	√	√
	BSA		VTK*CCTESLVNR			√
2	HSA	K549	K*QTALVELVK	√		
	RSA		K*QTALVELVK	√	√	√
	BSA		K*QTALVELLK	√	√	√
3	HSA	K249	FPK*AEFAEVSK	√	√	
	RSA		FPK*ADFTDISK	√	√	
	BSA		FPK*AEFVEVTK	√	√	√
4	HSA	Y108	ETY*GEMADCCAK	√	√	√
	RSA		DTY*GDVADCCEK	√	√	√
	BSA		ETY*GDMADCCEK	√	√	√

The active sites of the phosphonylated adducts were mainly distributed in the various amino acid residues with active side chains, while the disulfide adduct sites were limited to cysteine residues without species-related differences. As shown in Table 5, a total of six disulfide adducts were identified between the V-type agents and HSA (four sites), or BSA (two sites). These peptides (VTKCCTESLVNR and ETYGEMADCCAK in HSA, ETYGDMADCCEK in BSA) were labeled at cysteine residues, and were regarded as potential biomarkers for V-type agents exposure through phosphonylation identification previously. These modified sites further confirmed the reliability of the modified peptides as potential biomarkers. However, there is no recognized disulfide adducts as biomarkers at present. This may be due to the formation mechanism of disulfide adducts. The disulfide adducts formed via disulfide bonds exchange between the thiol groups from the leaving groups and cysteine residues, following the formation of phosphonylation adducts^39,53^. The data in Table 5 showed that the labeled peptides from the disulfide adducts were accompanied by phosphonylated sites, which further verified this conclusion.

**Table 5:** Disulfide adducts in different species after V-type OPNAs exposure.

Modified peptide	Phosphonylated sites			Disulfide sites			Species
	VX	Vs	VR	VX	Vs	VR	
VTK*C*C*TESLVNR	K499	K499	K499		C500 C501	C500 C501	human
QNC*ELFEQLGEY*K	Y424		Y424	C416	C416	C416	human
ETY*GEMADC*CAK	Y108	Y108	Y108	C114			human
ETY*GDMADC*CEK	Y108	Y108	Y108		C114	C114	bovine
SLHTLFGDELC*K		C99	C99	C99		C99	bovine

### MS/MS analysis of the modified peptides

**Figure 2:**
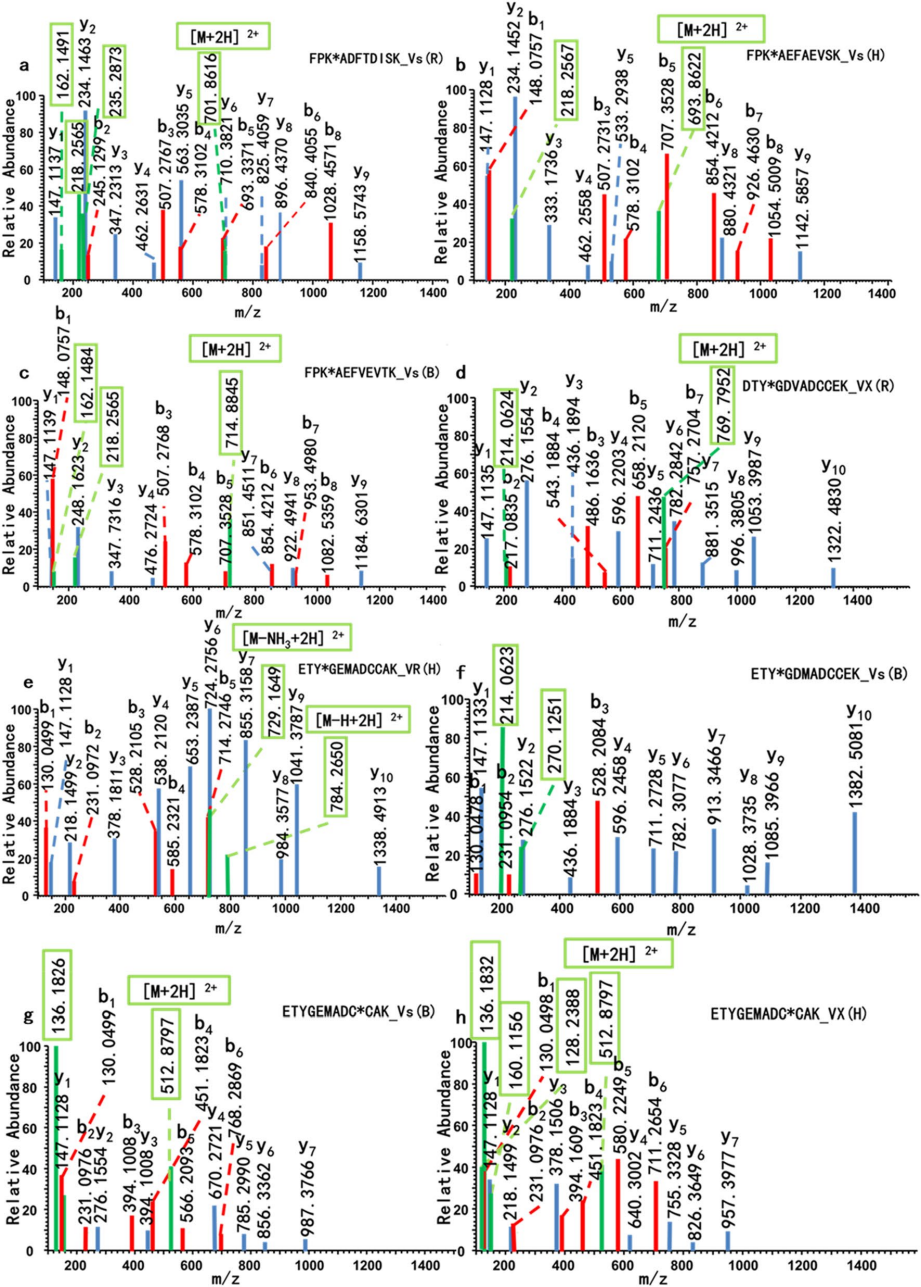
MS/MS spectra of labeled peptides in albumins after V-type OPNAs exposure. (**a**–**c**) showed K249 on FPK*ADFTDISK in RSA, FPK*AEFAEVSK in HSA and FPK*AEFVEVTK in BSA all phosphonylated by Vs. (**d**–**f**) showed Y108 on ETY*GDVADCCEK in RSA, ETY*GEMADCCAK in HSA and ETY*GDMADCCEK in BSA phosphonylated by VX, VR, Vs, respectively. (**g**–**h**) showed C114 on ETYGDMADC*CEK in BSA and ETY*GEMADCCAK in HSA were binding to Vs and VX, respectively. Key peaks marked in blue lines for y-ion series, red lines for b-ion series and green lines for characteristic precursor ions.

Mass spectrometry had served as a powerful tool for identifying protein adducts, and it can provide reliable evidence to further explore the adduct mechanism and discover more active sites^18,25,50,54–56^. The OPNA-albumin adducts formed when small molecules attacked the active residues in the albumin. Some stable and reliable adducts were regarded as potential biomarkers that can be utilized in detecting OPNA exposure. The accurate molecular weight of the amino acid residues of trypsin-digested peptides was determined before and after OPNAs modification. Thus, the reactive sites were discovered by calculating the mass-to-charge ratio of b and y series ions. The representative MS/MS spectra of highly reactive peptides in albumins from different species were shown in Fig. 2, where the key peaks were marked by blue lines for y-ion series, red lines for b-ion series, and green lines for the characteristic precursor ions.

Figure 2a–c showed MS/MS spectra of the Vs-K249 adducts in different serum albumins. The mass values of the parent ions were 701.8616 amu in RSA, 693.8622 amu in HSA and 714.8845 amu in BSA in the form of [M + 2H]^2+^. These results represented the mass of the unlabeled peptide plus the mass of the O-butyl methylphophonate group (134.0497 amu), which belonged to Vs and one proton, and reflected phosphonylated peptides by Vs. The characteristic fragments ions of the V-type OPNAs adduct were presented in Table 6. The characteristic precursor ions were captured, including phosphoryl lysine imine ions losing ammonia, its fragment ion after losing the butyl group and the phosphoryl lysine imine ions, which provided direct evidence for phosphonylation at lysine residues. The characteristic ions fragments from albumin adducts at lysine were summarized in Table 7. Two lysine residues in the entire peptide were potential phosphonylation sites, but more effective signals are needed to determine the position of phosphonylated residue. The mass of the y1 ion equaled the mass of lysine residue indicating that it had not been modified and modified site may be at the other lysine residue. The y1–y8 ions did not show phosphonylation of the AEFAEVSK amino acid sequences, while the mass gap between y8 and y9 ions equaled the mass of the lysine residue plus one molecule of O-butyl methylphophonate. This demonstrated that Vs was bound to K249 on FPK*AEFAEVSK. This conclusion was further confirmed by the corresponding mass differences between the b2 and b3 ions.

**Table 6:** Characteristic ions of the structure of V-type OPNAs in albumin adducts.

OPNAs	Phosphonylation adducts	m/z (amu)	Disulfide adducts	m/z (amu)
VR		134.0497		131.0769
Vs		134.0497		131.0769
VX		106.0184		159.1082

**Table 7:** Characteristic ions of albumin adducts at lysine residue.

OPNAs	Structure	m/z (amu)	Characteristic ion
VR		218.2568	Phosphoryl lysine imine ion -NH3
		162.1488	Aging phosphoryl lysine imine ion -NH3
		235.2878	Phosphoryl lysine imine ion
		179.1798	Aging phosphoryl lysine imine ion
Vs		218.2568	Phosphoryl lysine imine ion -NH3
		162.1488	Aging phosphoryl lysine imine ion -NH3
		235.2878	Phosphoryl lysine imine ion
		179.1798	Aging phosphoryl lysine imine ion
VX		190.2028	Phosphoryl lysine imine ion -NH3
		162.1488	Aging phosphoryl lysine imine ion -NH3
		207.2338	Phosphoryl lysine imine ion
		179.1798	Aging phosphoryl lysine imine ion

Figure 2d–f showed the phosphonylated tyrosine adducts (VX-Y108 in RSA, VR-Y108 in HSA, and Vs-Y108 in BSA). The parent ion fragments, including [M + 2H]^2+^ (769.7952 amu) (Fig. 2d), [M-H + 2H]^2+^ (784.2650 amu) and [M-NH_3_ + 2H]^2+^ (729.1649amu) (Fig. 2e), helped to confirm the phosphonylated peptides and modified small molecules, while no relevant information was found in Fig. 2f. The characteristic ion peaks of the methyl phosphonate tyrosine imine ion (214.0624 amu) indicated that this peptide was modified by V-type agents, and the O-alkylphosphonate tyrosine imine ion (270.1251 amu) provided the evidence of phosphonylated by Vs or VR^30,39^. The b and y series ions assisted in offering accurate information that further confirmed site modification. The mass gap between the b_3_ and b_2_ ions was equal to the mass of one tyrosine residue (163.0633 amu) plus the O-alkylphosphonate group. The mass of the peptide GEMADCCAK corresponded to the y_1_-y_9_ ions, while the mass differences between the y_9_ and y_10_ ions equaled the b_3_ ion minus the b_2_ ion. It provided the evidence for phosphonylated site Y108 at the third residue from the C-terminus in the ETY*GDMADCCEK peptide.

**Figure 3:**
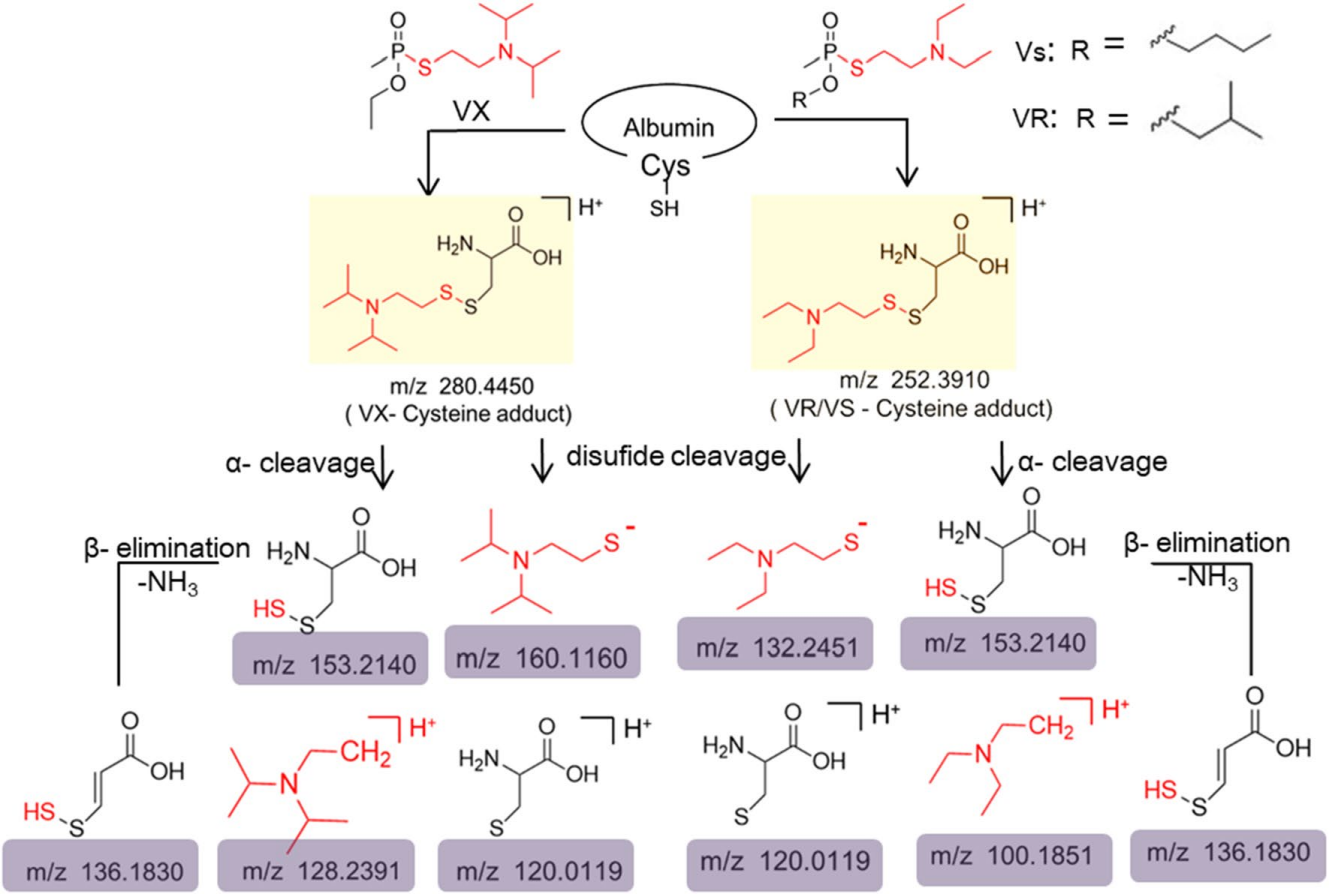
Characteristic ions of disulfide adducts after V-type OPNAs exposure.

Figure 2g–h showed the disulfide adducts between the cysteine residues and Vs in BSA (Fig. 2g), VX in HSA (Fig. 2h). The characteristic ion peaks of the leaving group of VX (2(diisopropylamino) ethanethiol) (*m/z* 160.1156), trimethylamine (*m/z* 128.2388) generated by α- cleavage, and (E)-3-disulfanylacrylic acid (*m/z* 136.1832) or (*m/z* 136.1826) generated by β-elimination. The characteristic ions of albumin-adducts at endogenous cysteine residues after V-type OPNAs exposure were summarized in Fig. 3. The mass values of the b_1_–b_6_ ions corresponding to ETYGEM from the N terminus, and y_1_-y_3_ corresponding to CAK from the C terminus offered evidence that there were no modification sites. The mass gap of y_4_ ions minus the y_3_ ions equaled the molecular weight of the cysteine residue plus the corresponding dialkylamino ethylthiol group of VX or Vs. Therefore, these data suggested that C114 on peptide ETYGDMADC*CEK was modified by the class V agents with a disulfide bond.

Liquid chromatography-tandem mass spectrometry was a commonly used method to detect modified peptides obtained by the enzymatic hydrolysis of protein adducts^9,26,28,48^. Analysis of the modified peptides was based mainly on the mass differences between the b and y series ions, and the characteristic fragment ions in secondary mass spectrometry. Characteristic ions consisting of the same structure, such as 2(diethylamino) ethanethiol) from VR or Vs, and isomers such as the O-butyl methylphophonate group from Vs and the O-isobutyl methylphophonate group from VR are not distinguished by mass spectrometry. In addition, according to the peak intensity of the disulfide adduct fragment ions and linker bond breakage energies, the possible characteristic fragment ions of the disulfide adducts were summarized in Fig. 3, and generated by disulfide bond breakage, α-cleavage and β-elimination.

### Stably labeled HSA peptides by OPNAs

The ability of the OPNAs to bind to amino acids in albumin was affected by the number of active residues^13,18,25,54,56^. One molecule of OPNAs is covalently linked to one molecule of active amino acid. Using tyrosine as an example, 609 amino acid residues were found in one molecule of albumin, including nineteen tyrosine residues. Ideally, all 19 tyrosine residues would be modified by 19 molecules of OPNAs. With a constant concentration of albumin, augmenting the concentration of OPNAs would increase the binding rate between the OPNAs and active residues until the modified active sites were saturated. Thus, the lower the concentration, the fewer active residues were available for OPNAs modification. The residues continuously modified were identified as stable modification sites, suggesting the high selectivity for V-type poisons and the potential as biomarkers.

**Figure 4:**
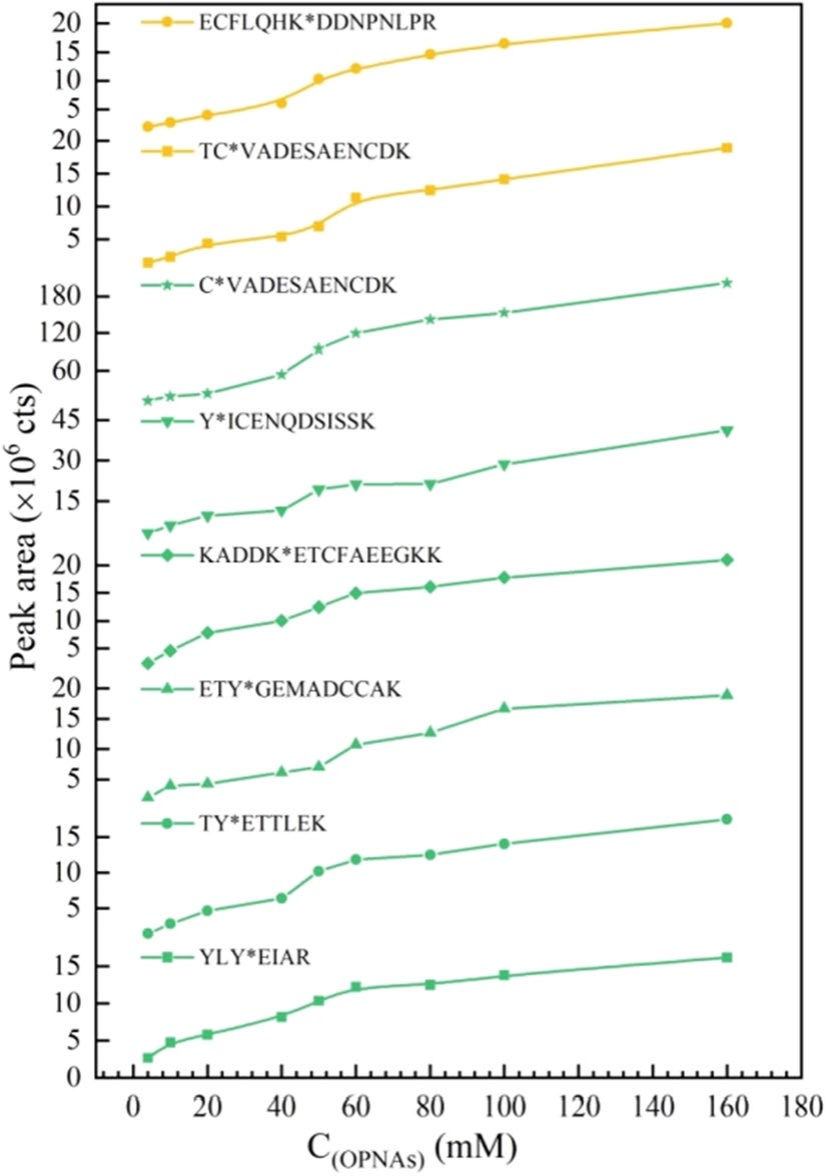
Peak area of peptides modified by V-type OPNAs with gradient concentration. C_(OPNAs)_ represents the final concentration in the solution system. Peptides Y*ICENQDSISSK, TY*ETTLEK, YLY*EIAR, KADDK*ETCFAEEGKK, C*VADESAENCDK and ETY*GEMADCCAK modified by VX in green. TC*VADESAENCDK and ECFLQHK*DDNPNLPR modified by Vs in yellow.

For HSA, seven peptides that could be stably modified by various concentrations of OPNAs were identified. As shown in Fig. 4, Gradient concentration of OPNAs refers to the final concentration of OPNAs in the solution system. The peak area of the phosphonylation sites was enhanced with increasing OPNAs concentration, and the increased signal intensities in the mass spectra indicated the binding rates of the OPNAs to these sites increased. As shown in Table 8, sustainable modification sites were observed in HSA exposed to V-type agents in a range of concentrations. Y108 on ETY*GEMADCCAK, Y287 on Y*ICENQDSISSK, Y377 on TY*ETTLEK, and Y164 on YLY*EIAR were stably phosphonylated by all of the poisons, providing evidence for identifying V-type OPNAs exposure as potential biomarkers.

**Table 8:** Stably labeled peptides in human albumin after V-type OPNAs exposure.

NO	Labeled peptide	Modified sites	VX	VR	Vs
1	Y*ICENQDSISSK	Y287	VX1	VR1	Vs1
2	TY*ETTLEK	Y377	VX1	VR1	Vs1
3	YLY*EIAR	Y164	VX1	VR1	Vs1
4	KADDK*ETCFAEEGKK	K588	VX1	VR2	Vs1
5	C*VADESAENCDK	C77	VX3		
6	TC*VADESAENCDK	C77			Vs3
7	ECFLQHK*DDNPNLPR	K130			Vs1
8	ETY*GEMADCCAK	Y108	VX1	VR1	Vs1

### Molecular simulation and structural analysis of the Vs adducts

Molecular simulation was used to study the protein adducts between the stable modification sites (K588, Y287, Y108, K130, Y164, Y377, and C77) and Vs by (1) calculating the solvent accessibility (SAS) and solvent accessible area ratios (% SAS) of the active sites (residue exposure in HSA when %SAS > 10) to determine the positions of modified residues on HSA, (2) predicting the interactions between the active sites and surrounding residues, and inferring the possible influencing factors to promote adduct formation, (3) predicting the covalent interactions between the potential biomarker and Vs, and understanding the influence of spatial effect of the active sites on HSA modification.

Table 9 showed that the five modified sites (K588, Y287, Y108, K130 and Y164) were in exposed state in the HSA structure, which increased the collision probability between these sites and Vs and provided better reactive conditions for adduct formation. The %SAS of the two residues Y377 and C77was approximately zero, which indicated they were buried in HSA. It is worth noting that the residues distributed on the surface of the HSA could increase the possibility of binding the small molecules of toxic agents, while the buried residues still had the ability to attack the phosphorus atoms in Vs.

**Table 9:** The solvent accessibility of labeled residues in HSA structure.

No	Residues	SAS (Å^2^)	%SAS	Exposed/buried
1	K588	169.493	87.158	Exposed
2	Y287	51.404	23.412	Exposed
3	Y108	29.696	13.525	Exposed
4	K130	19.951	10.259	Exposed
5	Y164	6.693	3.048	Buried
6	Y377	0	0	Buried
7	C77	0	0	Buried

The interactions between the active sites and surrounding residues could create powerful conditions for adduct formation between HSA and Vs. Two types of modified site interaction were found, including hydrogen and disulfide bonds, as shown in Table 10. Hydrogen bonding forces increased with decreasing distance between the labeled sites and others. Generally, hydrogen bonding action is effective within a distance of 3.5 Å. We observed that Y287, K130, Y164, and Y164 formed strong hydrogen bonds with the corresponding residues as the hydrogen bond distances were all within 2.5 Å. The strongest interactions were found between the Y164 (acceptor) and R192 (donor). These interactions exhibited a certain hydrophobicity, which helped to stabilize the toxic molecules bound to the active sites. C77 also displayed a disulfide bond with C110 at a distance of 2 Å. The side chain of C77 will be in a bound state under normal conditions, and may become free during the experiment. Subsequently, disulfide bond was formed with Vs. C77 is a potential biomarker as it was stably modified under these conditions. The binding free energy of Vs to the active sites was also calculated. The lower binding free energy resulted in the stronger binding action between Vs and HSA, providing the greater probability of Vs binding to the active sites. The binding energies of these active sites were similar. K130 had the lowest binding energy with Vs, and creating advantageous conditions for subsequent covalent binding.

**Table 10:** The interaction among labeled residues and Vs.

Residues	E_GBSA_ (kcal/mol)	Interaction	Interaction type	Interaction sites	Distance(Å)
K588	− 19.5087	**–**	**–**	**–**	**–**
Y287	− 23.7176	Hydrogen Bond	H-Acceptor	N315	2
Y108	− 26.6937	–	–	–	–
K130	− 30.8920	Hydrogen Bond	H-Donor	P195	2.3
Y164	− 29.9765	Hydrogen Bond	H-Acceptor	R192	1.9
Y377	− 23.4347	Hydrogen Bond	H-Acceptor	L405	2.3
C77	− 22.6775	Disulfide Bond		C110	2

**Figure 5:**
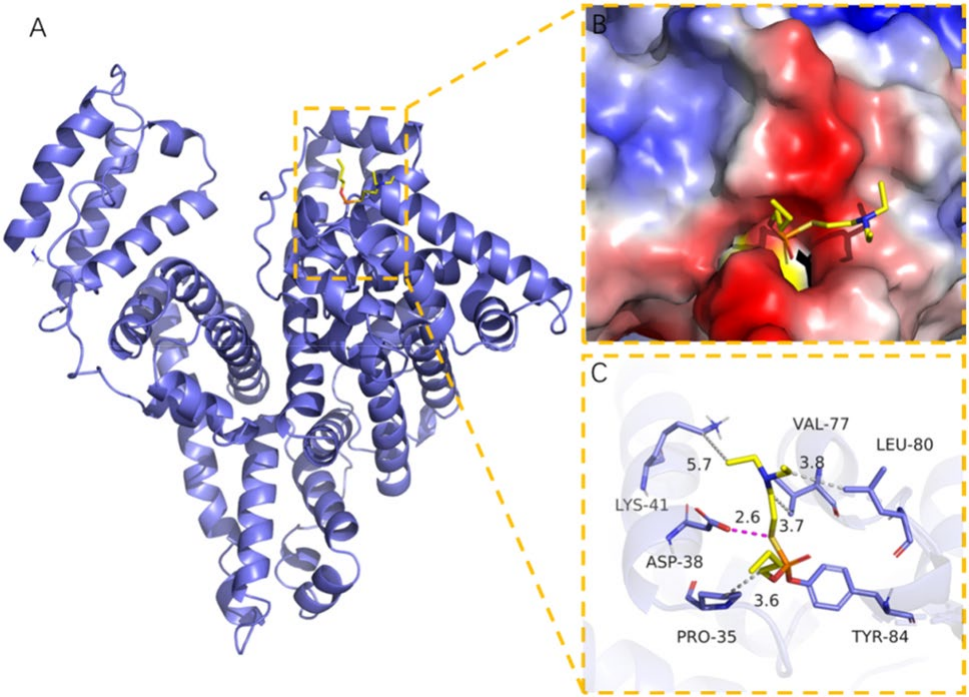
Detailed binding mode of Vs adducts in HSA. A was 3D structure of HSA, B showed VDM surface of binding site, C was interaction among Vs and active site residues. Residue number form PDB.

The ETY*GEMADCCAK peptide was determined to be stably modified by V-type agents, where Y108 on the peptide was regarded as a potential biomarker for detecting OPNA exposure. As shown in Fig. 5, covalent molecular docking was used to predict the covalent binding energy between Vs and active residue Y108 (Y84). The results showed lower binding energy (− 5.51 kcal/mol, less than standard value − 5 kcal/mol) between Vs and the labeled site, illustrating there is a certain binding ability and good matching degree. To obtain a binding pattern between Vs and HSA, these adducts were visualized with Pymol 2.1 software. According to the binding patterns, the amino acid residues in the HSA pocket and Vs were observed, as well as the interaction between Vs and other residues (V77, L80, P35, K41, and D38) adjacent to Y108 was found, as shown in Table 11. This adduct formed by phosphorus groups from Vs covalently linked to the hydroxyl group at Y108. The hydrophobic chains with certain hydrophobicity formed hydrogen bonds with V77, L80, P35, and K41 in the active pocket of albumin. Thus, the hydrophobic effect of hydrogen bonds played an important role in stabilizing the small molecules of the toxic agents in HSA. In addition, electrostatic repulsions between the sulfur atoms of Vs and the carboxyl groups of D62 also contributed to stabilizing Vs binding to the active site. In summary, Vs had a good binding ability and matching degree with Y108 in the HSA.

**Table 11:** The promoting factors for the formation of adduct between Vs and Y108.

Target	OPNAs	Binding energy (kcal/mol)	Residues	Combination type
HSA	Vs	− 5.51	Y108(Y84)	Covalent bond
			V101(V77)	Hydrogen Bond
			L104(L80)	Hydrogen Bond
			P59 (P35)	Hydrogen Bond
			K65(K41)	Hydrogen Bond
			D62(D38)	Electrostatic interaction

Residue number is from Uniprot website, number in brackets is from PDB, with a difference of 24.

## Conclusions

This work took HSA, BSA and RSA as research objectives, discovering the possible biomarkers and expanding the group of the possible biomarkers in different species, which provided a theoretical basis for retrospective identifying potential biomarkers to determine V-type OPNAs exposure. The possible active residues in albumins were examined according to the following two parallel experimental workflows: neat HSA, BSA, and RSA were sequentially exposed to V-type OPNAs in vitro, and pure HSA was subjected to class V agents in different concentrations.

Numerous shared active sites were observed in albumins from different species including phosphonylation sites (four sites) and one disulfide adduct site. Notably, Y108 on ETY*GEMADC*CAK in HSA, DTY*GDVADC*CEK in RSA, and ETY*GDMADC*CEK in BSA were shared phosphonylation site labeled by all poisons tested. C114, a disulfide adduct site on these homologous sequences, was selectively modified by class V agents in both HSA and BSA. The results indicated that these sequences could be used as potential biomarkers to trace the exposure of V-type OPNAs. In particular, Y108 on ETY*GEMADC*CAK in HSA was stably phosphonylated by V-type agents in gradient concentration which further illustrated the reliability of this sequence for screening V-type OPNAs. Molecular docking showed the promoting factors for the formation of Vs adducts at Y108 in HSA consisted of low covalent binding energy, hydrogen bonding, and electrostatic interactions among the residues adjacent to Y108 and Vs. Y287 on Y*ICENQDSISSK, Y377 on TY*ETTLEK and Y164 on YLY*EIAR in HSA were also stable phosphonylated by all the poisons tested. This suggested they were potential biomarkers of V-type toxicant exposure as well.

## Materials and methods

Caution: V-type OPNAs are highly toxic organophosphorus compounds. To ensure their safety, the operators were professionally trained and protected.

### Materials

V-type OPNAs (> 97% pure) were synthesized at the Analytical Chemistry Laboratory in the Institute of Chemical Defense (Beijing, China). Purified HSA, RSA and BSA were purchased from Sigma-Aldrich (St Louis, MO, USA). Dithiothreitol (DTT), iodoacetamide (IAM), Tris–HCl buffer (1 M, pH 7.4), and MD10 bag filters (8000–14000D) were provided by Solarbio Science and Technology Company (Beijing, China). Ammonium hydrogen carbonate (NH_4_HCO_3_) was supplied from the Shanghai Macklin Biochemical and Technology Company (Shanghai, China). Sequencing-grade trypsin was purchased from Promega (Madison, WI, USA), and ultrafiltration tubes (UFC5010BK) were purchased from Merck-Millipore (Shanghai, China). All other reagents (analytical grade) were purchased from Beijing Chemical Works (Beijing, China).

### V-type OPNAs working solution

VX, Vs, and VR were dissolved in isopropanol to obtain 10 mg/mL solution that was stored in a refrigerator at 4 °C.

### Sample preparation for modified peptide segments

Albumin (200µL, 1 mg/mL) was incubated with a 20-fold molar excess of 15µL V-type agents isopropanol solution in a Tris–HCl buffer (0.1 M, pH 7.4) at 37 °C, overnight. Then, the protein was denatured in a water bath at 95 °C for 20 min. The obtained products were reduced by adding 0.25 mL of DTT (10 mM DTT in 25 mM NH_4_HCO_3_ solution) at 57 °C for 1 h, and alkylated using 0.25 ml of IAM (50 mM DTT in 25 mM of NH_4_HCO_3_ solution) at room temperature for 1 h in the dark. The mixture was dialyzed with a 100-fold volume of 10 mM NH_4_HCO_3_ solution overnight to remove the excess agent molecules, and the dialysate was changed twice. The dialyzed samples were mixed with 0.1 mL trypsin (20 ng/mL of trypsin in 25 mM NH_4_HCO_3_ solution) with a volume ratio of 4:1 and incubated at 37 °C for 16 h. The undigested protein was removed using 0.5 mL 10 kDa ultrafiltration tubes at 14,800 rpm for 15 min. The digested peptides were combined and stored at – 20 °C for future use^28–30,33,35,39^.

### Dose–response of albumin peptides

A 200µL HSA solution (1 mg/mL of HSA in 0.1 M Tris–HCl buffer at pH 7.4) was mixed with 15 µL of V-type OPNAs (i.e. VX, Vs, VR) isopropanol solution, yielding molar ratios of HSA to OPNAs of 1:20, 1:50, 1:100, 1:200, 1:250, 1:300, 1:400, 1:500, and 1:800. Subsequently, the mixtures were prepared according to the above experimental procedures.

### LC–MS analysis

The peptide mixtures were analyzed on an Orbitrap Fusion Lumos (Thermo Fisher Scientific) mass spectrometer interface using an Easy-nLC 1200 nanoflow liquid chromatography system (Thermo Fisher Scientific) with nanospray ionization (NSI) in positive ion polarity mode. The samples were dissolved in mobile phase A (0.1% formic acid aqueous solution) and mixed thoroughly. Then, Nano LC system (Thermo Fisher Scientific) with a 150 μm × 12 cm C18 reversed-phase chromatographic column (2 cm × 100 µm) was used to separate the samples. The mobile phase consisted of phase A (0.1% formic acid and 100% water) and phase B (0.1% formic acid, 80% acetonitrile and 20% water). The elution program was set to a 30 min linear gradient elution (25 min 11% B, 1 min 41% B and 4 min 100% B) and the flow rate was 600nL/min. Data acquisition adopt Orbitrap fusion Lumos mass spectrometer with nano-current electrospray ion source (NSI), the spray voltage of 2200 V and Ion transport capillary of 320 °C. Mass spectrometry data were collected in data-dependent acquisition (DDA) mode in the positive ion mode. A full scan (200–1500 m/z, 120,000 resolution) was conducted via an Orbitrap mass analyzer. Related parameters included the scanning range of 200–1500 m/z, the resolution of 120,000, the automatic gain control targets of 5e^5^ ions and iron max injection time of 50 ms. The fragment ions were detected in the Orbitrap following fragmentation of the parent ions via 32% high-energy collision dissociation using two-dimensional mass spectrometry. Related parameters included the first mass of 50, the resolution of 150,000, the automatic gain control targets of 5e^5^ ions and iron max injection time of 22 ms.

### Qualitative analysis of protein

The original mass spectral data were searched with Sequest, which was embedded in Proteome Discoverer 2.4. HSA, RSA, and BSA sequence databases were obtained from Uniprot. The parameters deviations of the parent ion and fragment ion mass were set to 20 ppm and 0.05 Da, respectively. False positives were controlled by reverse sequence decoy strategy, and the results were verified using Percolator software.

### Computer simulations

Molecular simulations were performed using Discovery Studio 4.5 molecular simulation software. The three-dimensional crystal structure of HSA was obtained from the protein crystal database (http://www.rcsb.org/pdb/, PDB number 1bm0). Residue solvent accessibility (SAS) and its percentage (%SAS) were obtained using the DelPhi method according to the electrostatic potential of the focused section. Then, the non-binding interaction monitor method was applied to predict non-binding interactions of the seven active residues with adjacent amino acids. A molecular docking technique was used to calculate the noncovalent binding energies between Vs and the modified residues in the HSA^28,33,39^.

Covalent docking between Vs and Y108 on HSA was conducted on the molecular manipulation platform (MOE2019.01). Protein processing and molecular docking were performed using the structure preparation and docking modules respectively. Afterward, the interactions between the surrounding residues adjacent to Y108 and Vs were calculated afterward.

## Data Availability

All data supporting the findings of this study are provided within the manuscript.
